# A systemic study on the vulnerability and fatality of prostate cancer patients towards COVID-19 through analysis of the *TMPRSS2*, *CXCL10* and their co-expressed genes

**DOI:** 10.5808/gi.22012

**Published:** 2022-09-30

**Authors:** Md. Thosif Raza, Shagufta Mizan

**Affiliations:** Department of Genetic Engineering and Biotechnology, Faculty of Biological Sciences, University of Chittagong, Chattogram 4331, Bangladesh

**Keywords:** COVID-19, *CXCL10*, prostate cancer, *TMPRSS2*

## Abstract

A pandemic of respiratory disease named coronavirus disease 2019 (COVID-19) is caused by a novel coronavirus, severe acute respiratory syndrome coronavirus 2 (SARS-CoV-2). It is reported prostate cancer patients are susceptible to COVID-19 infection. To understand the possible causes of prostate cancer patients' increased vulnerability and mortality from COVID-19 infection, we focused on the two most important agents, transmembrane protease serine subtype 2 (*TMPRSS2*) and the C-X-C motif 10 (*CXCL10*). When SARS-CoV-2 binds to the host cell via S protein‒angiotensin-converting enzyme-2 receptor interaction, *TMPRSS2* contributes in the proteolytic cleavage of the S protein, allowing the viral and cellular membranes to fuse. *CXCL10* is a cytokine found in elevated level in both COVID-19 and cancer-causing cytokine storm. We discovered that *TMPRSS2* and *CXCL10* are overexpressed in prostate cancer and COVID-19 using the UALCAN and GEPIA2 datasets. The functional importance of *TMPRSS2* and *CXCL10* in prostate cancer development was then determined by analyzing the frequency of genetic changes in their amino acid sequences using the cBioPortal online portal. Finally, we used the PANTHER database to examine the pathology of the targeted genes. We observed that *TMPRSS2* and *CXCL10*, together with their often co-expressed genes, are important in the binding activity and immune responses in prostate cancer and COVID-19 infection, respectively. Finally, we found that *TMPRSS2* and *CXCL10* are two putative biomarkers responsible for the increased vulnerability and fatality of prostate cancer patients to COVID-19.

## Introduction

Since its emergence from Wuhan (Hubei, China) [1], to date, the severe acute respiratory syndrome coronavirus 2 (SARS-CoV-2) has been held accountable for millions of deaths and billions of active cases globally [2]. The causative agent of coronavirus disease 2019 (COVID-19), SARS-CoV-2 is one of many pathogens of zoonotic origin [3] that were able to direct cross-species transmission, driving low to severe health complications in masses, leading to epidemics and pandemics [4]. Coronaviruses (CoVs) have been infecting humans since the 1960s, with alphacoronaviruses HCoV-229E and HCoV-OC43 dominating the infections. Previous studies have found that bats and rodents are the most notable hosts to zoonotic viruses [5,6]. Bats, particularly, are a favorable host for CoVs, with 31% of the bat viromes being constituted by CoVs [7-9]. Previous epidemics and pandemics that resulted from zoonotic spillover caused deaths from hundreds to millions in number. Given the severity of the clinical and social burden epidemics and pandemics impose, studying the underlying mechanisms of zoonosis in detail is crucial [10,11].

Comorbidity, as a risk factor for COVID-19 has been accentuated in multiple clinical and epidemiological studies [1,12-14]. Multiple comorbid conditions, such as cardiovascular diseases, diabetes, chronic kidney diseases, hypertension, respiratory diseases and cancer make individuals vulnerable to both pathogenic and non-pathogenic diseases as their immunity system is compromised. Often, certain metabolic pathways, genes and proteins are upregulated in individuals with cancer, which is favorable for opportunistic pathogens, allowing them to multiply at an amplified rate in host cells [15].

During SARS-CoV-2’s life cycle within a host, a number of biomolecules play both active and passive roles in promoting its pathogenesis. The fundamental molecule in this case is the angiotensin-converting enzyme-2 (*ACE2*) receptor, which the virus utilizes to harbor its spike protein for entering inside the host cell. However, the virus cannot fuse with the host cell’s membrane unless the spike (S) protein undergoes proteolytic priming [16,17]. When SARS-CoV-2 anchors itself to the host cell with the help of S protein-*ACE2* receptor binding, a type II transmembrane serine protease called transmembrane protease serine subtype 2 (*TMPRSS2*) participates in the proteolytic cleavage of the S protein, facilitating the fusion of the viral membrane and the cellular membrane [18]. Another group of molecules that act as key indicators during the pathogenesis are cytokines [19]. When SARS-CoV-2 invades a host cell, the host’s innate immune response is activated. The cells of the innate immune system act using pattern recognition receptors (PRRs). PRRs detect pathogen associated molecular patterns which are distinctive to SARS-CoV-2 and deploy inflammatory responses against the invading virus by activating a cascade of signaling pathways and transcription factors [20]. These signaling pathways activate three pro-inflammatory cytokines interleukin (IL)-1, tumor necrosis factor (TNF)-α, and IL-6, which are upregulated to such an extent that the effect of this upregulation becomes debilitating for the host, resulting in tissue damage, multi-organ failure and often, death [21-23]. According to a study, the C-X-C motif 10 (*CXCL10*), or Interferon gamma-induced protein 10 is a notable cytokine molecule in the prognosis of COVID-19, which is capable of causing severe tissue damage and is also involved in pathological processes of infectious diseases [24,25].

As the number of COVID-19 active cases and deaths started to peak, a sex bias was observed in the prevalence rate as well as the mortality rates [26,27]. Multiple studies conducted in different countries and populations reported that males are at a higher risk of being infected with SARS-CoV-2 and some studies linked this higher prevalence with that of prostate cancer [28,29]. Prostate cancer or prostate adenocarcinoma (PRAD) is the second most frequently occurring cancer among males, with the most recent epidemiology stating a total 1,276,106 new cases and 358,989 deaths (which is 3.8% of all deaths caused by cancer in men) [30]. Individuals with prostate cancer are at no lesser risk of developing a severe clinical prognosis of COVID-19 than other cancer types. The *ACE2* receptor is not only unique to lung cells, but they are also found in the kidneys, prostate and intestine [31]. The presence of the *ACE2* receptor in other organs suggests a possibility of SARS-CoV-2’s metastasis and localization in other cells. Moreover, the androgen receptor is a key transcription factor of *TMPRSS2*, which is upregulated in the presence of testosterone. The *TMPRSS2* protease is also found to be upregulated in both normal and metastatic cancer cells [32-34]. As for the chemokine molecules, studies support that *CXCL10* is associated with exacerbating inflammation and also plays a role in the pathological process of cancers [24,25]. Based on these facts, it is plausible to state that because *ACE2*, *TMPRSS2*, and *CXCL10* are commonly contributing molecules in both the pathogenesis of SARS-CoV-2 and PRAD, men could be more susceptible to acquiring COVID-19.

In this study, we used a computational approach to investigate the expression patterns, molecular and functional characterization of *CXCL10* and *TMPRSS2* and their coexpressed genes in PRAD and COVID-19. The study was carried out *in silico*, using existing, open access cancer omics databases and bioinformatics tools whose algorithms have been upgraded to provide well-grounded results based on real scientific evidence of clinical value. Two similar studies carried out by Hoang et al. [35] and Kalkanli et al. [36] have also performed an assessment of the susceptibility of PRAD patients towards COVID-19. However, these two studies were focused towards the expression analysis of *ACE2* and *TMPRSS2* rather than *CXCL10*, which is an important inflammatory cytokine that plays an active role in cancer metastasis [37-39]. Moreover, we also analyzed the commonly coexpressed genes in both PRAD and COVID-19 to identify common pathways leading to a severe prognosis of COVID-19 in PRAD patients. We compared the expression patterns of the aforementioned genes in both normal cells and cancer cells to truly assess the differences in the expression levels; and gauge the degree of risk prostate cancer patients are at when it comes to being infected by SARS-CoV-2 and developing COVID-19 associated health complications.

## Methods

### Expression analysis of *TMPRSS2* and *CXCL10* in PRAD

For assessing the magnitude of mRNA expression of *TMPRSS2* and *CXCL10* genes in PRAD, we used TIMER 2.0 web server (http://timer.comp-genomics.org/). TIMER 2.0 implements six robust algorithms to expression profiles of tumors obtained from The Cancer Genome Atlas (TCGA), which is an upgrade to its previous single algorithm version to analyze and compare immune infiltrates in tumor cells and normal cells [40]. To further investigate the expression profiles of *TMPRSS2* and *CXCL10*, UALCAN web server was used (http://ualcan.path.uab.edu/). UALCAN web server allows comprehensive analysis of a target gene expression in tumor cells and normal cells using real time data from a range of clinical profiles. The analyses are conducted to provide insights on the variation of clinicopathologic features based on race, sex, ethnicity as well as stages in a particular type of cancer [41]. GEPIA2 (http://gepia2.cancer-pku.cn/#index) web server was used to compare the median expressions of *CXCL10* and *TMPRSS2* genes between normal and tumor samples of PRAD. GEPIA2 processes query data using expression profiles obtained from TCGA and GTEx databases and returns gene specific analyses based on multiple cancer types [42].

### Determination of mutations and copy number alterations in *TMPRSS2* and *CXCL10*

We used cBio Cancer Genomics Portal (https://www.cbioportal.org/) to identify genetic alterations of *TMPRSS2* and *CXCL10* genes and analyse their molecular and clinical profiles. cBioPortal curates data from large scale cancer genomics projects, providing a substantial amount of data on molecular profiles of cell lines and cancer tissues that can be translated into facts of biological and clinical significance [43].

### Protein-protein interaction network construction

We prepared a list of determinant genes of the clinical prognosis of COVID-19 using Comparative Toxicogenomics Database (CTD, http://ctdbase.org/). The CTD database is a centralized resource that collects data from valid and proven scientific studies and presents the relationship of specific chemicals, genes and proteins with a myriad of diseases and disorders [44]. Upon preparing a list of genes that have a remarkable contribution in the clinical prognosis of COVID-19, we analyzed the interaction between the protein products of these genes using the STRING database (https://string-db.org/). The STRING database constructs a protein-protein interaction network between targeted proteins by interpreting protein-protein associated data that are either known or predicted in a large number of organisms. The reliability and authenticity of the generated physical and functional interactions of the proteins in question are annotated using confidence scores and the evidences supporting the results are traceable [45].

### Identification of coexpressed genes

We used R2: Genomics and Visualization platform (https://hgserver1.amc.nl/cgi-bin/r2/main.cgi) an open access omics database to identify the commonly expressed genes in correlation with *TMPRSS2* and *CXCL10*. This database includes data from different biomedical analyses that can be used for enhanced molecular analysis of target genes in regards to multiple diseases producing insights of clinical value [46]. A Venn diagram was generated using the Bioinformatics and Evolutionary Genomics (http://bioinformatics.psb.ugent.be/webtools/Venn/) web tool to represent the identified coexpressed genes of *TMPRSS2* and *CXCL10* in COVID-19 and PRAD.

## Results

### *TMPRSS2* and *CXCL10* expression in prostate cancer

**Fig. 1.**
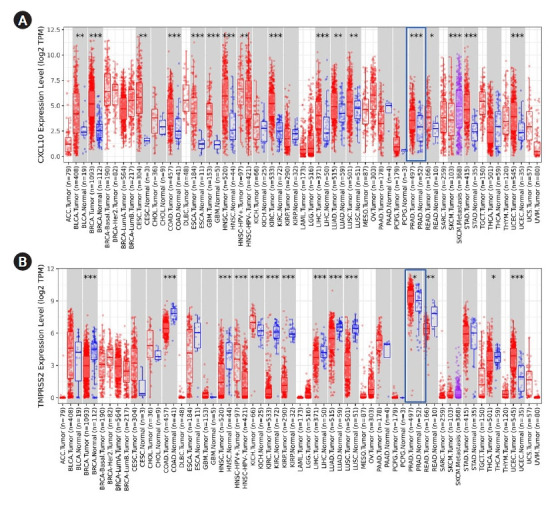
Analysis of expression of *CXCL10* and *TMPRSS2* in different cancer by using TIMER. (A) Different level of *CXCL10* mRNA expression is shown in multiple cancer studies whereas the overexpression of *CXCL10* in PRAD, is marked in the red box. (B) Different level of *TMPRSS2* mRNA expression is shown in multiple cancer studies whereas the overexpression of *TMPRSS2* in PRAD is marked in the red box. *p < 0.05, **p < 0.01, ***p < 0.001. *CXCL10*, C-X-C motif 10; *TMPRSS2*, transmembrane protease serine subtype 2; ACC, adrenocortical carcinoma; BLCA, bladder urothelial carcinoma; BRCA, breast invasiva carcinoma; CESC, cervical squamous cell carcinoma; CHOL, cholangiocarcinoma; COAD, colon adenocarcinoma; DLBC, diffuse Large B-cell Lymphoma; ESCA, esophageal carcinoma; GBM, glioblastoma multiforme; HNSC, head and neck squamous cell carcinoma; HPV, human papillomavirus; KICH, kidney chromophobe; KIRC, kidney clear cell carcinoma; KIRP, kidney renal papillary cell carcinoma; LAML, acute myeloid leukemia; LGG, brain lower grade glioma; LIHC, liver hepatocellular carcinoma; LUAD, lung adenocarcinoma; LUSC, lung squamous cell carcinoma; MESO, mesothelioma; OV, ovarian serous cystadenocarcinoma; PAAD, pancreatic adenocarcinoma; PCPG, pheochromocytoma and paraganglioma; PRAD, prostate adenocarcinoma; READ, rectum adenocarcinoma; SARC, sarcoma; SKCM, skin cutaneous melanoma; STAD, stomach adenocarcinoma; TGCT, testicular r germ cell tumors; THCA, thyroid carcinoma; THYM, thymoma; UCEC, uterine corpus endometrial carcinoma; UCS, uterine carcinosarcoma; UVM, uveal melanoma.

To start, first different expression patterns of *TMPRSS2* and *CXCL10* in multidisciplinary cancer types were analyzed using the TIMER database. Both *TMPRSS2* and *CXCL10* showed an escalated pattern of expression in PRAD compared to normal tissue (Fig. 1). p-value for *TMPRSS2* gene was <0.05 whereas *CXCL10* gene showed p-value <0.001 in the PRAD patients. *TMPRSS2* manifested the highest level of expression in PRAD than any other cancer tissue.

### Analysis of *TMPRSS2* and *CXCL10* expression pattern in PRAD

**Fig. 2.**
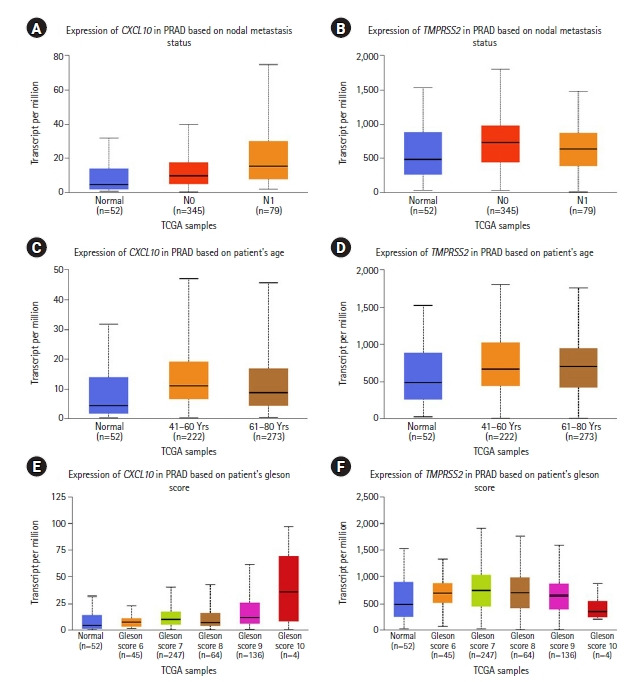
Expression analysis of *CXCL10* and *TMPRSS2* in prostate cancer using the UALCAN database. (A) *CXCL10* gene expression in PRAD based on different nodal metastasis status. (B) *TMPRSS2* gene expression in PRAD based on different nodal metastasis status. (C) *CXCL10* gene expression in PRAD according to the different age groups. (D) Expression of the *TMPRSS2* gene in PRAD according to different age groups. (E) Expression of the *CXCL10* gene in in PRAD in different cancer stages based on Gleason score. (F) *TMPRSS2* gene expression in PRAD in different cancer stages based on Gleason score. *CXCL10*, C-X-C motif 10; *TMPRSS2*, transmembrane protease serine subtype 2; PRAD, prostate adenocarcinoma; TCGA, The Cancer Genome Atlas.

We performed an extensive analysis to evaluate the association of *CXCL10* and *TMPRSS2* expression with multiple clinicopathological parameters using the TCGA dataset retrieved from the UALCAN data mining platform. Here, we found an overall upregulated expression pattern of *CXCL10* and *TMPRSS2* compared to a normal state depending on the individual stages of cancer and different age groups (Fig. 2). In terms of nodal metastasis status, both genes showed higher expression in PRAD patients with N0 and N1 stages compared to normal individuals. For *CXCL10*, the highest expression was found at N1 stage while the expression of *TMPRSS2* peaked at N0 stage. Patients with PRAD showed the highest level of *CXCL10* expression at age between 41 and 60 years whereas prostate cancer patients at age 61-80 years showed highest level of expression for *TMPRSS2*. PRAD patients with Gleason score 10 showed the highest level of expression for *CXCL10*. On the other hand, expression of *TMPRSS2* peaked in patients having Gleason score 7.

### Determination of mutations and copy number alterations in target proteins

**Fig. 3.**
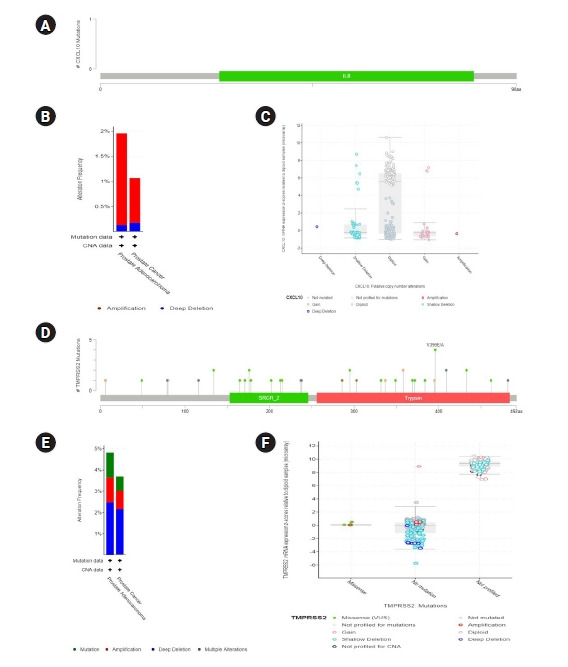
The functional characterization of *CXCL10* and *TMPRSS2* in prostate cancer development by using the cBioPortal database using 5,584 samples of 5,389 prostate cancer patients from 18 studies. (A) Mutations in CXCL10 protein sequence was figured out by using lollipop plots. (B) Types of alteration frequencies of *CXCL10* in prostate cancer were presented in bar diagram. (C) The expression level of differently categorized genetic alterations was presented for *CXCL10*. (D) Total mutation in TMPRSS2 protein was presented by lollipop plots. (E) Three variant types of alteration frequency in *TMPRSS2* were presented in the bar diagrams. (F) The expression level regarding the multiple categories of genetic alteration was represented in graphical plots based on Z-scores relative to diploid samples. *CXCL10*, C-X-C motif 10; *TMPRSS2*, transmembrane protease serine subtype 2; PRAD, prostate adenocarcinoma; CNA, copy number alteration; VUS, variant of uncertain significance.

Data generation was done representing multiple genetic variations in *TMPRSS2* and *CXCL10* mRNA using the cBioPortal database to assess the functional significance of *TMPRSS2* and *CXCL10* in prostate cancer development (Fig. 3). Firstly, we prepared a query for *CXCL10* in this database using 5,584 samples of 5,389 prostate cancer patients from 18 studies. From this analysis, we found no mutations for *CXCL10* in PRAD patients. We observed that *CXCL10* is mostly altered in PRAD ranging the highest frequency of 1.82%. In this case, we found that the highest level of CNA occurred due to the diploid type of genetic alteration. The second most significant genetic change is done by shallow deletion type of CNA. From our investigation it was found that 47 mutations occurred for *TMPRSS2* in prostate cancer patients (Supplementary Table 1). *TMPRSS2* showed most genetic alterations in prostate adenocarcinoma with alterations frequency of 4.81%. Forty-seven mutations were found in 30 locations of *TMPRSS2* protein in prostate cancer patients. Shallow deletion accounted for the second most genetic alterations of *TMPRSS2* in prostate cancer while most alterations did not account for mutation.

### Association of *TMPRSS2* and *CXCL10* assisted protein-protein interaction network with the of COVID-19 development

**Fig. 4.**
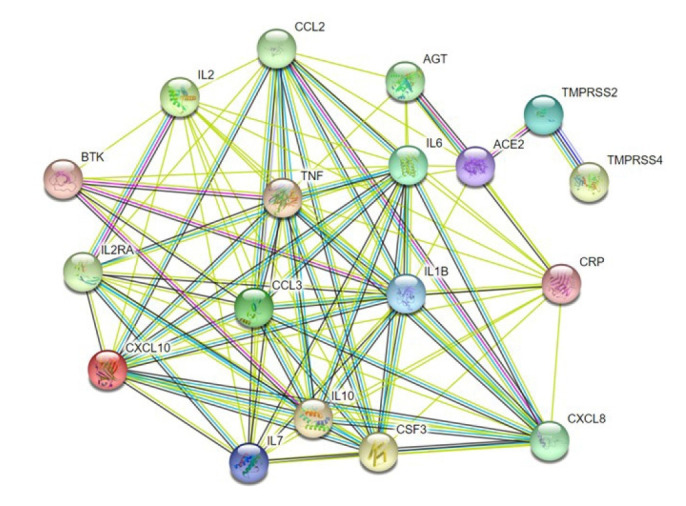
A protein-protein interaction network illustrating the interconnection of the functional proteins related to COVID-19 infection. *TMPRSS2* is mainly interconnected with ACE2 and TMPRSS4 whereas *CXCL10* is interlinked with other cytokines such as IL6, IL7, TNF, and IL10. Other proteins have several cross-linked connections as well. *CXCL10*, C-X-C motif 10; ACE2, angiotensin converting enzyme 2; AGT, angiotensin; COVID-19, coronavirus disease 2019; CXCL8, C-X-C motif 8; IL6, interleukin 6; *TMPRSS2*, transmembrane protease, serine 2; TMPRSS4, transmembrane protease, serine 4; IL2, interleukin 2; IL2RA, interleukin 2 receptor alpha chain; CCL2, C-C motif ligand 2; TNF, tumor necrosis factor; IL10, interleukin 10; CSF3, colony stimulating factor; CRP, C-reactive protein; IL1B, interleukin 1 beta; CCL3, C-C motif ligand 3; IL7, interleukin 7; BTK, Bruton's tyrosine kinase.

COVID19 is caused by a number of genes that are either directly or indirectly involved in its development. Based on their inference score, we were able to discover 7,235 genes related with COVID-19 disease using the CTD. Each of these genes has a curated illness relationship or an assumed disease association based on a curated chemical interaction. Following the analysis of the large data collection, 20 curated genes were identified as biomarkers or therapeutic candidates for COVID-19 therapy, including *TMPRSS2* and *CXCL10* (Supplementary Table 2). By utilizing the translated protein sequences of these 20 genes a protein-protein interaction network was constructed through the STRING database (Fig. 4). Following this, we found 96 connecting edges among the selected proteins though the expected edges were only 20 according to the information provided by the database itself. That means the PPI network harbors more interlinks than the expected result. Such a relationship indicates that the proteins are functionally connected, as a group. We also found that *TMPRSS2* and *CXCL10* proteins are interconnected along with other protein components associated with COVID-19 development.

### Estimation of the commonly co-expressed genes of *TMPRSS2* and *CXCL10* associated with prostate cancer and COVID-19 development

**Fig. 5.**
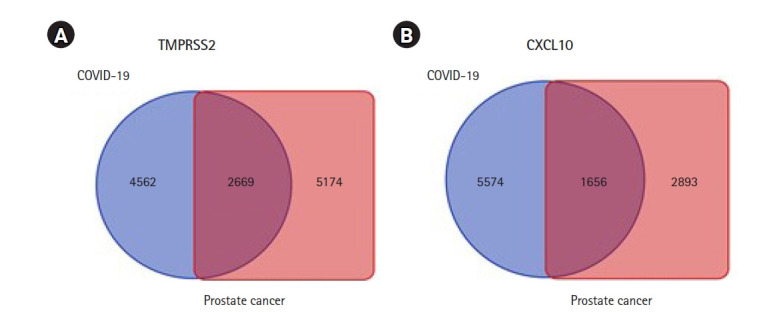
Graphical representation of commonly co-expressed genes of *TMPRSS2* and *CXCL10* in prostate cancer and COVID-19. Identification of co-expressed genes of *TMPRSS2* and *CXCL10* was completed through a comprehensive analysis by using the R2: Genomics and Visualization web portal. (A) The Venn diagram represents 2,669 commonly co-expressed genes of *TMPRSS2*. (B) The Venn diagram represents 1,656 commonly co-expressed genes of *CXCL10* associated with both of the diseases. *TMPRSS2*, transmembrane protease serine subtype 2; *CXCL10*, C-X-C motif 10; COVID-19, coronavirus disease 2019.

Identification of genes that tied in with the expression of *TMPRSS2* and *CXCL10* was completed through a comprehensive analysis by using the R2: Genomics and Visualization web portal. This identification assisted in exploring the co-expressed genes of *TMPRSS2* and *CXCL10* responsible for prostate cancer and COVID-19 development by utilizing the TCGA data. In particular, a total of 7,843 co-expressed genes of the *TMPRSS2* associated with PRAD whereas the number of co-expressed genes related to COVID-19 was 7,231. In terms of *CXCL10*, 4,549 genes happened to co-express with prostate cancer development while those with COVID-19 were 7,230. A restriction of p-value < 0.01 was applied to each case of the analysis. Afterward Venn diagrams were contrived using the Bioinformatics and Evolutionary Genomics web tool in order to show the lists representing common co-expressed genes of *TMPRSS2* and *CXCL10* in each of the cases of prostate cancer and COVID-19. Overall, 1,656 and 2,669 genes co-expressed with *CXCL10* and *TMPRSS2* respectively, were found to be common prostate cancer and COVID-19 progression (Fig. 5).

### Analysis of the functional role of the *TMPRSS2* and *CXCL10*

**Fig. 6.**
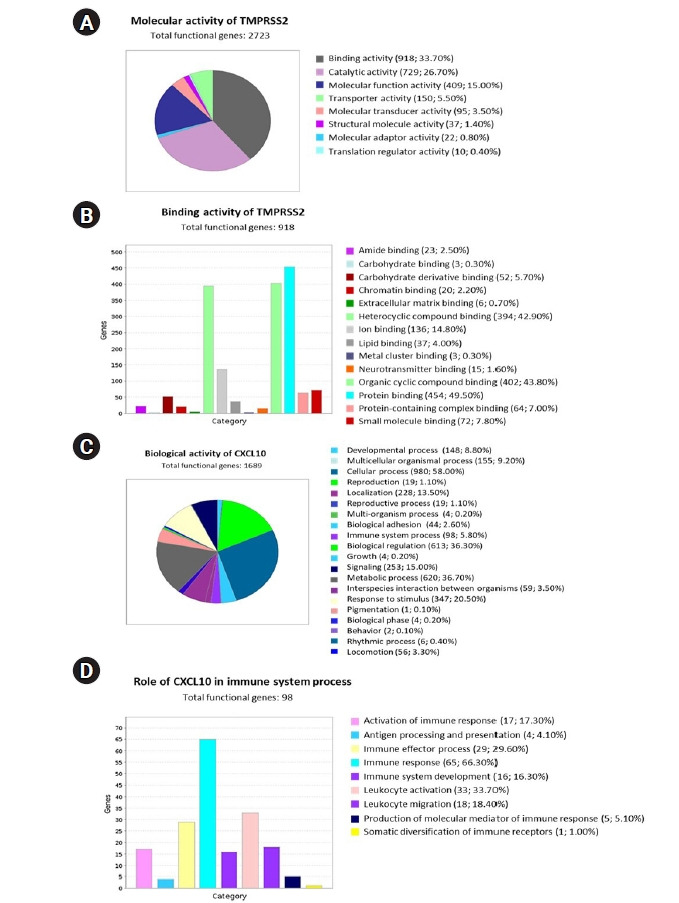
Using the PANTHER database, the functional attitudes of *TMPRSS2* and *CXCL10* were evaluated. (A) A pie chart was used to show eight different types of molecular activity of *TMPRSS2* and its co-expressed genes. (B) In total 14 variant types of binding activities of 918 genes were represented by using a bar chart. (C) Total 20 unique types of biological activities of *CXCL10* and its co-expressed genes were represented using a pie chart. (D) Nine differently categorized immune system processes of the corresponding 98 co-expressed genes were presented through a bar chart. *TMPRSS2*, transmembrane protease serine subtype 2; *CXCL10*, C-X-C motif 10.

To interpret the functional activity of *TMPRSS2* and *CXCL10* in prostate cancer and COVID-19, we used the set of previously determined co-expressed genes while using the PANTHER database. To begin, we ran a query to determine the molecular activity of *TMPRSS2* by using the 2,669 commonly co-expressed genes that had previously been found. We looked at a variety of molecular activities and observed that a large number of these genes (918) are engaged in binding activity, whereas 26.70% (729) are involved in catalytic activity (Fig. 6A). We observed the diverse nature of the binding activities of corresponding 918 genes through an extended study. Following this analysis, we observed that most of the genes having binding activity are involved in protein binding (49.50%; 454 genes) (Fig. 6B).

We also investigated the functional attitude of *CXCL10* using the list of previously determined 1,656 commonly co-expressed genes which have association with prostate cancer and COVID-19 development. We found that the mentioned genes are engaged in a wide range of biological activities, including cellular processes, biological regulation, metabolic processes, immune system processes, localization, biogenesis, and so on, after evaluating the biological processes (Fig. 6C). After performing an extended analysis with the co-expressed genes of *CXCL10* involved in different immune processes, we explored that 65 genes are involved in immune response, 16 genes are responsible for immune system development, 33 genes are responsible for leukocyte activation whereas 18 genes are responsible for leukocyte migration, 17 genes contribute to the activation of immune response and 29 genes lead the immune effector process (Fig. 6D). Overall, a significant number of the co-expressed genes of *CXCL10* are found to have important roles in various branches of the immune system along with the functional activity of *CXCL10*.

## Discussion

Since the emergence of COVID-19 and its outspread globally, mortality rates have been more inclined towards the male population as compared to the female population, which has been established by multiple epidemiological studies [47-49]. Severe clinical prognosis of COVID-19 has also been attributed to comorbidities, with special emphasis on the presence of cancer [50-52]. PRAD is a prevalent cancer type in males, and multiple biomolecules that act as markers of PRAD are in common with COVID-19 (Both *TMPRSS2* and *CXCL10* were upregulated in PRAD patients). *TMPRSS2* has been reported as a prominent transmembrane protein that is upregulated in PRAD patients [28]. The upregulation of this protein has also been found to be a crucial factor for *ACE2* priming, which is the major receptor for SARS-CoV-2 entry. Cytokine storms have been a widely discussed phenomenon in regards to both COVID-19 and cancer [53,54]. *CXCL10* is a notable cytokine, which has been found in elevated levels in both cancer and COVID-19 patients, driving metastasis, inflammation and poor clinical outcomes in both the cases [55,56]. A study by Gwak et al. [57] on the prostate cancer microenvironment found that multiple inflammatory cytokines including *CXCL10* were upregulated in PRAD patients. *CXCL10* plays a significant role in the tumor metastasis and immunesuppression in prostate cancer and being a tumor-associated macrophage, it also promotes tumor microenvironment creation, migration and invasion of prostate cancer cells [37-39]. Thus, in the case of both PRAD and COVID-19, *TMPRSS2* and *CXCL10* can be considered as two prominent biomarkers. In this study, we analyzed the expression levels of *TMPRSS2* and *CXCL10* in PRAD patients. We found significant expressions of mRNA for both *TMPRSS2* (p < 0.05) and *CXCL10* (p < 0.001) in PRAD patients with higher levels as compared to normal individuals. Additionally, the demographic expression data revealed that both *TMPRSS2* and *CXCL10* were upregulated in PRAD patients aged 41‒60 years and 61‒80 years respectively with Gleason scores 7 to 9. These results align with a study conducted by Chen et al. [58], which found a significant upregulation of *TMPRSS2* in patients aged 40‒65 years having a median gleason score of 7 to 9 but patients with advanced stage like metastasis did not show considerable expression change. Although adults aged 65 or more have been identified to be more susceptible towards SARS-CoV-2 infection [59], people aged 60 years and less are at no lesser risk. In a recent cross-sectional survey conducted in various countries of Europe and America, it was found that 45‒22% of COVID-19 associated fatalities were more prevalent in individuals aged less than 65 years [60]. According to a report by the American Cancer Society, PRAD is more prevalent in males aged 65 or more, which is also the risk cohort for COVID-19. In a study conducted by Peng et al. [61], an increase in *TMPRSS2* expressions in oral epithelial cells has been found with age. A study by Schuler et al. [62] has also found elevated expressions of *TMPRSS2* in lung epithelium of adults. *TMPRSS2* is also expressed on the luminal side of the normal prostatic epithelium, which is upregulated in malignant prostatic tissue [29]. Hence, the expression analysis data of *TMPRSS2* and *CXCL10* aligns with the findings of previous studies.

For genomic analyses of *TMPRSS2* and *CXCL10* coding genes, we scrutinized whether mutations were present in *TMPRSS2* and *CXCL10* in PRAD patients. The reference database consisted of data obtained from 18 different studies on PRAD. We found the highest level of alterations in *TMPRSS2* (4.81% alteration frequency) and a total of 47 mutations were observed. A 1.82% alteration frequency was also observed for *CXCL10* in PRAD patients. Genetic alterations in PRAD patients have been found to be related to the disparity among prostate cancer patients globally. Genomic aberrations, gene expression signature, and other molecular alterations in tumors have also led to variation in disease progression in PRAD patients, leading to distinct pathways based on clinical heterogeneity. These genomic alterations can happen at any stage of the cancer progression, yielding three possible molecular subtypes of prostate cancer: (1) clinically localized, treatment naïve prostate cancer, (2) aggressive metastatic but, hormone sensitive prostate cancer, and (3) lethal, androgen deprivation therapy insensitive, castration resistant prostate cancer [63]. Analysis of CNAs in *TMPRSS2* and *CXCL10* may help in assessing the clinical features of COVID-19 patients with aggressive or indolent prostate cancer. It may also assist in better understanding immunological pathways that are triggered in different subtypes of prostate cancer in presence of SARS-CoV-2 providing better opportunities for specific treatment and management of COVID-19 in this cohort. In addition, deletions, CNAs and other types of genetic modifications in active biomolecules can also contribute to understanding the significance of these molecules in disease development even among different populations [64].

As clinical manifestations in the cases of both COVID-19 and PRAD may metastasize and localize in other organs and systems, we carried out a functional assessment of *TMPRSS2* and *CXCL10* to identify what additional genes and their protein products are related to COVID-19 and PRAD prognosis. Based on inference score, we selected 20 such genes that are involved in the prognosis of both COVID-19 and PRAD. Multiple cytokines were found to have higher inference scores, which supports the fact that the cytokine storm is mediated by multiple chemokines [55].

In this study, we identified 1,656 genes co-expressed with *CXCL10* in both PRAD and COVID-19 and 2,669 genes co-expressed with *TMPRSS2* for the same. To properly understand the association of *TMPRSS2* and *CXCL10* with respect to the coexpressed genes and how these genes may contribute in disease prognosis, we selected 20 genes based on their inference scores established a PPI network between these two proteins and the translation products of the 20 genes. PPI network analysis tools utilize state-of-the-art algorithms and data from peer-reviewed research articles to illustrate the connection between proteins for better interpretation of underlying biological mechanisms for different diseases. PPI network analysis has been widely used in multiple cancer studies as it eases the interpretation of protein matrices on a molecular level [65-67]. From the PPI network analysis results, it is evident that *TMPRSS2* is a crucial factor in COVID-19 prognosis, as its nodes connect to *ACE2* and as per previous studies, *TMPRSS2* is a notable *ACE2* primer. This also suggests that because *TMPRSS2* is already upregulated in PRAD patients, the patients show increased susceptibility towards SARS-CoV-2 infection as an upregulation of *TMPRSS2* will promote *ACE2* priming [28,68,69]. Additional nodes were also found that connected *CXCL10* with other cytokines such as IL-6, IL-7, TNF, and IL-10. This finding aligns with previous reports that have observed that severely ill COVID-19 patients had elevated levels of IL-6 [22]. Our findings also align with the results of a study conducted on 41 COVID-19 confirmed cases in China, which found that IL-6, IL-7, TNF, and IL-10 were upregulated in the plasma of the study subjects [19]. Although *CXCL10* shows no evident interconnection with *TMPRSS2* in the PPI network, in PRAD patients, it has been linked to a group of tumor associated macrophages (TAMs) which are further classified into two different subtypes, M1 and M2. TNF-α, interferon γ, IL-12, and IL-23 comprise the M1 TAMs that have been found to have pro-inflammatory functions. IL-1β, IL-6, CXCL8, CXCL10, and vascular endothelial growth factor comprise the M2 TAMs that contribute to cancer metastasis, immune suppression, and tumor growth. Moreover, elevated levels of CXCL8, CCL2, CXCL10, and CCL20 have been found in prostate cancer patients with Gleason score ≥ 8 [57]. Again, COVID-19 patients too have upregulated levels of inflammatory cytokines which makes the disease prognosis critical in patients. Based on these facts, it can be suggested that coexpressed genes of *TMPRSS2* and *CXCL10* in both PRAD and COVID-19 play an active role in mediating the pathways responsible for disease prognosis.

The coexpressed genes were then further characterized functionally and it was found that of the 2,669 genes that were co-expressed with *TMPRSS2*, 33.70% were associated with binding activity, while 26.70% had involvement in catalytic activity. Further breakdown of the 33.70% genes associated with binding activity revealed that 49.50% of these genes are involved in protein binding. These findings can be supported by the fact that in PRAD cases, upregulation of *TMPRSS2* facilitates more activation of *ACE2* receptors which further makes a host more susceptible to SARS-CoV-2 infection. On the other hand, an extensive analysis of *CXCL10* co-expressed genes reported the association of multiple genes with immune response stages, including activation and development of immune response, leukocyte activation, leukocyte migration and immune effector processes. Noteworthy to mention that a full-fledged immune reaction is deployed against SARS-CoV-2 invasion as a consequence of an inflammatory trigger, and each step in this reaction requires the involvement of multiple immune cells and chemical responders. *CXCL10* is a key responder as it also attracts as a chemoattractant for immune cells like monocytes, T cells, and natural killer cells. In both cases of cancer and COVID-19, *CXCL10* exacerbates inflammation that causes tissue damage. In severe cases, cytokine storms accompanied by other chemokines due to severe inflammatory response can become fatal [25,70]. In regards to our study, as the findings indicate that the association of the co-expressed genes that take part in different immune response mechanisms are common to both PRAD and COVID-19, it can be predicted that PRAD patients, as they have higher levels of *CXCL10* (Fig. 2A), if diagnosed with COVID-19 have a possibility of developing cytokine storm mediated fatality.

This study was conducted *in silico* through utilization of different omics databases and genome analysis tools to identify how much of a threat COVID-19 is to patients with prostate cancer. Overall, we analyzed the expression patterns of two important determinant proteins of PRAD and COVID-19, *TMPRSS2* and *CXCL10*; scrutinized how the expression levels compare between age groups and explored the functional characteristics of associated genes of *TMPRSS2* and *CXCL10*. The data obtained from our analyses and previous relevant findings suggest that PRAD patients can be placed within the risk group of COVID-19 susceptibility and fatality.

Biological processes are regulated through the interaction of a myriad of chemicals and biomolecules that form perpetually complex meshes of pathways. These pathways become even more complicated in a state of infection or disorder. Often, early diagnosis of certain diseases cannot be carried out using conventional diagnostic tools. However, if certain biomarkers for these diseases can be identified, early prevention, treatment and management of diseases can be possible in different risk groups. Based on our *in silico* analysis, it can be said that *TMPRSS2* and *CXCL10* can serve as biomarkers for analyzing the susceptibility of prostate cancer patients towards COVID-19. The findings of this study can be utilized to identify possible associated proteins that also participate in the pathophysiology of PRAD and COVID-19, which can be used as targets for development of therapeutics directed towards prostate cancer patients. Although this *in silico* study presents substantial data that supports *TMPRSS2* and *CXCL10*’s association with COVID-19 susceptibility in prostate cancer patients, further experiments are required for deeper understanding of co-expressed genes, protein networks and host genetics that contribute to the polymorphism of *TMPRSS2* and *CXCL10* in individuals.

## Supplementary Materials

Supplementary Table 1. A list of genetic alterations in *TMPRSS2* protein sequences associated with prostrate cancer development.

Supplementary Table 2. The List of curated genes directly associated with COVID-19 development.
