# Supplementary material for: A systemic study on the vulnerability and fatality of prostate cancer patients towards COVID-19 through analysis of the TMPRSS2, CXCL10 and their co-expressed genes
Source: Genomics Inform. 2022 Sep 30;20(3):e31. doi: 10.5808/gi.22012 (PMC9576478; doi:10.5808/gi.22012)
Supplement: Supplementary Table 1. — A list of genetic alterations in TMPRSS2 protein sequences associated with prostrate cancer development. [file gi-22012suppl1.pdf]

Supplementary file 1

Table 1. A list of genetic alterations in *TMPRSS2* protein sequences associated with prostrate cancer development.

**Study of Origin**	**Sample Size**	**Protein Change**	**Mutation Type**	**Sample ID**
Metastatic castration-sensitive prostate cancer (MSK, Clin Cancer Res 2020)	424	R202K<br>W520Gfs*40	Missense_Mutation<br>Frame_Shift_Ins	P-0026237-T01-IM6<br>P-0022252-T01-IM6
Metastatic Prostate Adenocarcinoma (SU2C/PCF Dream Team, PNAS 2019)	444	V396E<br>W176C<br>D134Y<br>V396A<br>M372V<br>X358_splice<br>X337_splice	Missense_Mutation<br>Missense_Mutation<br>Missense_Mutation<br>Missense_Mutation<br>Missense_Mutation<br>Splice_Region<br>Splice_Region	PROS01448-6115247-Tumor-SM-67ES2<br>PROS01448-6115247-Tumor-SM-67ES2<br>MO_1184-Tumor<br>PROS11496-6115321-Tumor-SM-6CNQ5<br>PROS11496-6115286-Tumor-SM-6CNPY<br>PM158-TM<br>PRAD-6115593.0-Tumor-SM-B2XRV

Metastatic Prostate Cancer (SU2C/PCF Dream Team, Cell 2015)	150	V396E<br>W176C<br>D134Y<br>W384C	Missense_Mutation<br>Missense_Mutation<br>Missense_Mutation<br>Missense_Mutation	6115247<br>6115247<br>MO_1184<br>SC_9089
Neuroendocrine Prostate Cancer (Multi-Institute, Nat Med 2016)	114	N358=	Splice_Region	WCMC158_1_C
Prostate Adenocarcinoma (CPC-GENE, Nature 2017)	477	E178K	Missense_Mutation	TCGA-CH-5740-01
Prostate Adenocarcinoma (Fred Hutchinson CRC, Nat Med 2016)	176	G295D<br>G295D<br>G295D	Missense_Mutation<br>Missense_Mutation<br>Missense_Mutation	05-165K5_LUNG<br>05-165M1_LN<br>05-165O_ADRENAL

Prostate Adenocarcinoma (MSK, Eur Urol 2020)	1465	V49M	Missense_Mutation	P-0010518-T01-IM5
		V396E	Missense_Mutation	P-0013503-T02-IM6
		W176C	Missense_Mutation	P-0013503-T02-IM6
		G462D	Missense_Mutation	P-0008592-T01-IM5
		R409Mfs*8	Frame_Shift_Del	P-0002439-T01-IM3
		X80_splice	Splice_Site	P-0009546-T01-IM5
		R165K	Missense_Mutation	P-0026237-T01-IM6
		X6_splice	Splice_Site	P-0013032-T02-IM6
		I286del	In_Frame_Del	P-0015597-T01-IM6
		V237Afs*63	Frame_Shift_Del	P-0015618-T01-IM6
		S238Lfs*5	Frame_Shift_Del	P-0016941-T01-IM6
		W483Gfs*40	Frame_Shift_Ins	P-0022252-T01-IM6
		M349I	Missense_Mutation	P-0021183-T01-IM6

Prostate Adenocarcinoma (MSKCC/DFCI, Nature Genetics 2018)	1013	N304del	In_Frame_Del	TCGA-V1-A9OX-01
		G295D	Missense_Mutation	05-165O_ADRENAL
		D171Y	Missense_Mutation	MO_1184
		X395_splice	Splice_Region	PM156
		V433E	Missense_Mutation	PROS01448-6115247-SM-67ES2
		W213C	Missense_Mutation	PROS01448-6115247-SM-67ES2
		M409V	Missense_Mutation	PROS11496-6115286-SM-6CNPY
		V433A	Missense_Mutation	PROS11496-6115321-SM-6CNQ5
		E215K	Missense_Mutation	TCGA-CH-5740-01
		I369N	Missense_Mutation	TCGA-EJ-7793-01
		S116*	Nonsense_Mutation	TCGA-HC-A8D0-01
Prostate Adenocarcinoma (TCGA, Cell 2015)	333	I332N	Missense_Mutation	TCGA-EJ-7793-01
		S79*	Nonsense_Mutation	TCGA-HC-A8D0-01

Prostate Cancer (MSKCC, JCO Precis Oncol 2017)	504	G462D	Missense_Mutation	P-0008592-T01-IM5
		V396A	Missense_Mutation	P-0001071-T01-IM3
		R409Mfs*8	Frame_Shift_Del	P-0002439-T01-IM3
