# Supplementary material for: A systemic study on the vulnerability and fatality of prostate cancer patients towards COVID-19 through analysis of the TMPRSS2, CXCL10 and their co-expressed genes
Source: Genomics Inform. 2022 Sep 30;20(3):e31. doi: 10.5808/gi.22012 (PMC9576478; doi:10.5808/gi.22012)
Supplement: Supplementary Table 2. — The List of curated genes directly associated with COVID-19 development. [file gi-22012suppl2.pdf]

Table 2. The List of curated genes directly associated with COVID-19 development.

**Name of gene**	**Curated relationship**	**Inference Score**
CCL2	Biomarker	34.37
TNF	Biomarker	30.81
IL6	Biomarker	28.12
CXCL8	Biomarker	27.31
IL1B	Biomarker	26.53
IL10	Biomarker	26.16
IL2	Biomarker	21.02
AGT	Biomarker	19.98
CCL3	Biomarker	13.94
CXCL10	Biomarker	13.69
IL7	Biomarker	9.34

ACE2	Biomarker and Therapeutic target	7.69
CSF3	Biomarker	7.09
CRP	Biomarker	6.88
BTK	Biomarker	6.84
TMPRSS2	Biomarker	5.56
IL2RA	Biomarker	5.23
TMPRSS4	Biomarker	3
TMEM106B	Biomarker	2.86
LZTFL1	Biomarker	2.82
